# Dependence of DNA length on binding affinity between TrpR and *trpO* of DNA

**DOI:** 10.1038/s41598-020-71598-3

**Published:** 2020-09-24

**Authors:** Nobuo Shimamoto, Mikito Toda, Shigetoshi Nara, Tamiki Komatsuzaki, Kiyoto Kamagata, Takashi Kinebuchi, Jun-ichi Tomizawa

**Affiliations:** 1 National Institute of Genetics, Mishima, 411-8540 Japan; 2 Research Group of Physics, Faculty Division of Natural Sciences, Nara Women’s University, Kita-Uoya-Nishimachi, Nara, 630-8506 Japan; 3 Department of Electrical and Electronic Engineering, Okayama University, Okayama, 700-8530 Japan; 4 Research Institute for Electronic Science, Hokkaido University, Kita 20 Nishi 10, Kita-Ku, Sapporo, 001-0020 Japan; 5 Institute for Chemical Reaction Design and Discovery (WPI-ICReDD), Hokkaido University, Kita 21 Nishi 10, Kita-ku, Sapporo, Hokkaido 001-0021 Japan; 6 Institute of Multidisciplinary Research for Advanced Materials, Tohoku University, Katahira 2-1-1, Aoba-ku, Sendai, 980-8577 Japan; 7 Present Address: Veritas Kitayama, 30-1-104 Minamishiba-Cho, Sakyo-ku, Kyoto, 606-0841 Japan; 8 Present Address: Olympus Corporation, Quality Assurance and Regulatory Affairs, 2951 Ishikawa-machi, Hachioji-shi, Tokyo, 192-8507 Japan

**Keywords:** Biophysics, Molecular biophysics, DNA-binding proteins, Biophysical chemistry

## Abstract

We scrutinize the length dependency of the binding affinity of bacterial repressor TrpR protein to *trpO* (specific site) on DNA. A footprinting experiment shows that the longer the DNA length, the larger the affinity of TrpR to the specific site on DNA. This effect termed “antenna effect” might be interpreted as follows: longer DNA provides higher probability for TrpR to access to the specific site aided by one-dimensional diffusion along the nonspecific sites of DNA. We show that, however, the antenna effect cannot be explained while detailed balance holds among three kinetic states, that is, free protein/DNA, nonspecific complexes, and specific complex. We propose a working hypothesis that slow degree(s) of freedom in the system switch(es) different potentials of mean force causing transitions among the three states. This results in a deviation from detailed balance on the switching timescale. We then derive a simple reaction diffusion/binding model that describes the antenna effect on TrpR binding to its target operator. Possible scenarios for such slow degree(s) of freedom in TrpR–DNA complex are addressed.

## Introduction

How proteins encounter and bind DNA is one of the most intriguing subjects in biology. An exceptional feature of DNA–protein interactions is that a DNA molecule has multiple sites for a protein binding, yielding various isomeric protein–DNA complexes. They are generally classified into two types: those formed at most DNA sites with similar affinities and those at limited DNA sites with far larger affinities^1^. The former are called nonspecific complexes and are weakly maintained by sequence-independent interactions such as electrostatic forces between DNA backbone and a protein. The latter are called specific complexes and are stabilized by additional non-covalent interactions between residues of DNA and the protein. The genetic commands are mostly performed by the specific complex. Two binding pathways are possible. In a direct pathway, proteins form the specific complex via direct binding from bulk. On the other hand, proteins bind to non-specific DNA, diffuse along DNA as nonspecific complexes, and the diffused proteins eventually form specific complexes with their specific DNA sites^2,3^. Nonspecific sites can mediate the binding between a protein in the bulk and a specific site, if they are located near the specific site.

Surby and Reich reported 20-fold enhanced affinity of a monomeric enzyme, *Eco*RI methyltransferase, when the DNA fragment harboring a single specific site is extended from 14 to 775 bp DNA^4^. By von Hippel’s and Lu’s groups, 10–1,000-fold enhancement of affinity was reported for LacI^5,6^. This huge enhancement was interpreted by the fact that a single tetrameric LacI molecule simultaneously binds to two additional minor operators with a relatively high affinity as well as the major *lacO1* operator. However, a 20-fold enhancement remained for DNA fragments that have only *lacO1*, a significant antenna effect^5^. Namely, as DNA length increases, the protein affinity to the target site gets larger, which was named ‘antenna effect’ representing that DNA fragments (antenna) harbor the target site (detector) to receive proteins (signal) and bring them to that site or that the long antenna of insects collecting information to carry it to the central nerve system^3,7^. Similarly, the binding of *E.coli* RNA polymerase to its strongest promoter, T7A1 promoter, was shown to depend on DNA length. The dependence was once interpreted to accelerate the association rate for specific complex formation by harboring the DNA sequence in the nonspecific complexes^8^. Later, the effect was considered not to be kinetic enhancement, but rather enhancement of the affinity at “equilibrium”, because the protein–DNA system was supposed to be “equilibrated” in the experimental condition^9^. The antenna effect is expected to be an important factor in enabling proteins to form the specific complex at their specific sites more efficiently in the presence of tremendous non-specific sites in a cell.

Despite increased examples of the antenna effect, the theoretical description faces difficulty. Protein affinity to DNA is defined and determined at thermal equilibrium. At this condition, detailed balance should hold, which requires equality between forward and reverse reaction flows in any pathway. If the association flow of a protein to a specific site is enhanced in longer DNA by one-dimensional diffusion along the DNA, the dissociation flow of proteins from the specific DNA site should be also enhanced. Furthermore, the binding affinity is uniquely determined by the free energy difference between the dissociated components and the complex. When detailed balance holds, the protein affinity for the specific site should be independent of DNA length, as shown later. This suggests deviation from detailed balance for those protein–DNA systems showing the antenna effect.

Here, we propose a working hypothesis that slow degrees of freedom in protein/DNA system exist to switch different reaction mechanisms or different potentials of mean force which makes the system deviate from detailed balance in the switching timescale even while each reaction system may attain equilibrium. We postulate multiple potentials of mean force, and show what kinds of topographical features of the multiple potentials give rise to a larger deviation from detailed balance in the timescale of switching. We term this hypothesis ‘chemical ratchet’ hereinafter. A ratchet model was originally presented by Smolchowski^10^, and was also discussed by Feynman^11^. The idea of ratchets has been applied to various biological processes^12–16^ where some macroscopic quantities are in non-equilibrium. van Kampen pointed out that the internal degrees of freedom may not reach equilibrium within the timescale of reactions^17^. However, this possibility has not been explored in the study of ratchets. As an example, we consider a chemical ratchet alternating between two different potentials of mean force for a protein–DNA system composed of three kinetic states, free proteins and DNA, nonspecific complexes, and the specific complex. When the alternations occur slower than equilibration timescales on the potentials, the overall system deviates from detailed balance among the three kinetic states.

In the following, we first present the experimental evidence of the antenna effect on *E. coli* repressor TrpR binding to its specific DNA *trpO*. Second, we examine a one-dimensional diffusion model along the nonspecific sites of DNA preceded by protein–DNA binding at the specific site at equilibrium, and show that the antenna effect cannot be predicted while detailed balance holds. Third, we propose the concept of chemical ratchet as a working hypothesis to make the system deviate from detailed balance at some timescale, and revise the reaction diffusion/binding model to represent the antenna effect on TrpR binding to *trpO* by indirectly reflecting the idea of chemical ratchet. Finally, we address possible scenarios for such slow degree(s) of freedom in TrpR–DNA complex. Supplementary Information(SI) contains a detailed discussion of the model and explains the concept of the chemical ratchet. The definitions and the dimensions of the parameters are listed in Table 1 of the SI.

## Results

### Experiments revealed that TrpR binding to *trpO* shows antenna effect of 10,000 fold

**Figure 1:**
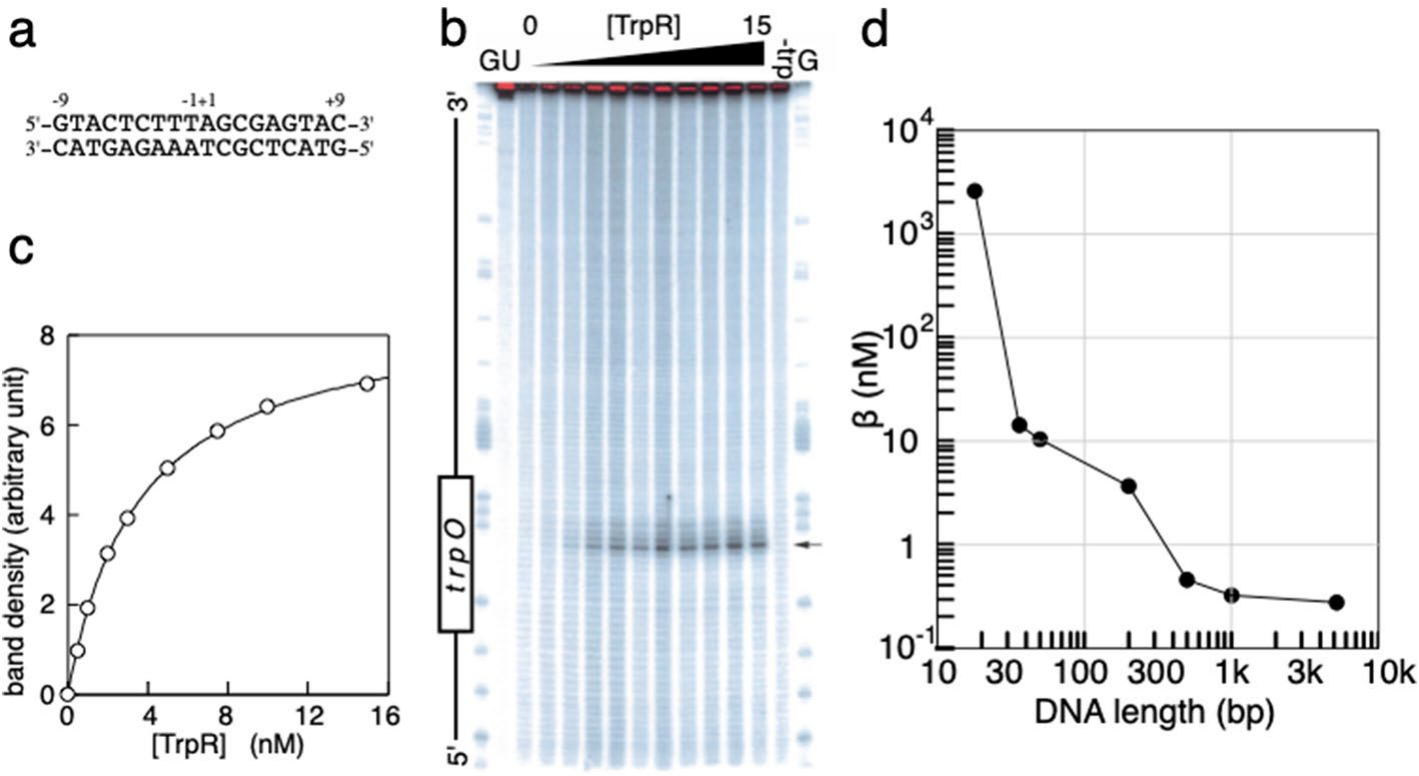
Hydroxide radical footprinting of TrpR binding to *trpO*. (**a**) The sequence of *trpO* of *trpR* gene^18^. DNAs of $18 \hbox {bp}{-}5.2 \, \hbox {kbp}$ long harbor this site *trpO* at their center. (**b**) Autoradiogram of the footprinting on 200 bp DNA. The cleavage reactions were performed in the presence of 0–15 nM TrpR with 5 mM L-tryptophan (triangle). The uncleaved DNA (lane U), cleaved but without TrpR (lane 0), and cleaved in the absence of corepressor L-tryptophan (–trp) were controls. Uncut DNA bands were over-exposed as shown in red in this autoradiogram, but total intensity of a lane were measured independently in a short exposure time. The position of *trpO* is indicated in the left, and the band most sensitive to the binding, ${-}$1T in the case of the upper strand, also shown by the arrow in the right. (**c**) The band intensities at various concentrations of TrpR. A measured band intensity was first normalized with the total intensity of the same lane for the correction of loading amount, and then the background intensity of no protein was subtracted from it. The intensities thus obtained are plotted against [TrpR] (open circle), and the least square fit to Eq. (1) (solid curve) gives the value of $\beta$. (**d**) The obtained values of $\beta$ plotted against DNA length showing 10,000-fold variation (see also Fig. 3).

We first examined the DNA length dependency (from 18 bp to 5.2 kbp) of the TrpR binding affinity to the specific site *trpO*. All the DNA harbor 18 bp *trpO* at their centers (Fig. 1a). The *trpO* of *trpR* gene^18^ was selected among the five operators in *E. coli* because it forms a one-to-one complex with a TrpR^19–21^. We conducted a hydroxyl radical footprinting^22^ for the binding assay to identify to which sequences along DNA TrpR binds at 1 bp spatial resolution. Briefly, a fixed amount of $^{32}$P-end labeled DNA fragment was pre-incubated with various excess amounts of TrpR to form TrpR-*trpO* complex for 2 h. Then, hydroxyl radical was added to cleave DNA at various points along the chain. If protein is bound to its specific site, the cleavage at that site is known to be enhanced^22,23^. The DNA fragments with different lengths were separated by electrophoresis in a sequencing polyacrylamide gel, and the radioactivity of individual fragments was detected by autoradiography. In Fig. 1b, uncut DNA contained trace amounts of DNA fragments as shown in the top half of the autoradiogram and the majority remained at the gel top as an over-exposed dense band (lane U). After the cleavage reaction, DNA was randomly cleaved by hydroxyl radical to generate uniform length distribution as shown in lane 0 where there exist no dense bands at single-cutting condition^23^. This condition enables us to quantify the statistics of cleavage of DNA of the same length. A similar uniform distribution was obtained in the absence of the physiological corepressor, L-tryptophan (lane-trp). In contrast, when L-tryptophan exists, a preferential cut was observed according to increasing concentration of TrpR as indicated by the dense and broad band. The location of the dense and broad band by referencing the G-ladder bands (lane G) corresponds to the specific binding to the *trpO* site. At several positions in the both strands within *trpO*, the cleavage was dependent on the concentration of TrpR but only with L-tryptophan. This is consistent with the fact that TrpR can form the specific complex only in the presence of L-tryptophan. Thus, the enhancement of the band intensity reflects the specific binding of TrpR to *trpO*. The intensities of the specific complex at each [TrpR] were well fitted to the equation expected for one-to-one binding (Fig. 1c):

1 $$\begin{aligned} ({\hbox {intensity at [TrpR]}}) - ({\hbox {intensity with no TrpR}}) = \frac{\alpha \,[{\hbox {TrpR}}]}{\beta + [{\hbox {TrpR}}]}, \end{aligned}$$

where $\alpha$ and $\beta$ are parameters independent of [TrpR], indicating the saturation level and the half-saturating [TrpR], respectively. As long as the left-hand side of Eq. (1) quantifies the concentration of TrpR-*trpO* complex, its saturation level is the total concentration of *trpO*. Thus, the left-hand side and $\alpha$ can be replaced by [TrpR-*trpO* complex] and [free *trpO*] + [TrpR-*trpO* complex], respectively, converting Eq. (1) into Eq. (2),

2 $$\begin{aligned} \beta \equiv \frac{[{\hbox {free}} \; trpO] [\hbox {TrpR}]}{[\hbox {TrpR-}trpO \; {\hbox {complex}}]}. \end{aligned}$$

Then, the parameter $\beta$ could to be called the dissociation (equilibrium) constant, $K_{\mathrm{d}}$, according to the mass-action law. In fact, this term has been used for the parameter corresponding to $\beta$^2,4–6,8,9^. However, this term is sometimes associated with implicit theoretical frameworks which must be examined in terms of detailed balance. Thus we reserve this expression until the examination. The obtained values of $\beta$ are plotted against DNA length in Fig. 1d.

### A theoretical model holding detailed balance does not show antenna effect

How can one rationalize the antenna effect of the protein–DNA system? Here we built a simple theoretical model specialized for the protein–DNA system and examined whether the model exhibits the antenna effect of TrpR. The model involves reaction processes composed of specific and nonspecific complexes and free proteins in the bulk as well as diffusion of protein along DNA. The concentration of the specific complex is denoted as $n_{\mathrm{S}}(t)$, and its dimension is [M]. On the other hand, we here assume “one-dimensional linear chain of DNA” specified by coordinate *x* with *trpO* at $x_S$ and “continuum approximation” in treating diffusion of protein along the DNA chain. Then, the concentration of nonspecific complex per a unit length at *x* at time *t* is denoted as $n_{\mathrm{NS}} (x,t)$, and its dimension is [$\hbox {M} \, {\mathrm{length}}^{-1}$]. Thus, $n_{\mathrm{NS}} (x,t)\Delta x$ denotes the concentration of nonspecific complex with protein located in the interval $[x,x+\Delta x]$. The concentrations of DNA with empty site at *x* is $n_{\mathrm{F}}^{\mathrm{DNA}} (x,t)=n_{\mathrm{tot}}^{\mathrm{DNA}} - n_{\mathrm{NS}} (x,t)\Delta d$ for $x\ne x_{\mathrm{S}}$ or $n_{\mathrm{F}}^{\mathrm{DNA}} (x_{\mathrm{S}},t)=n_{\mathrm{tot}}^{\mathrm{DNA}}- n_{\mathrm{S}}(t) - n_{\mathrm{NS}} (x_{\mathrm{S}},t)\Delta d$, where $n_{\mathrm{tot}}^{\mathrm{DNA}}$ is the concentration of total DNA, and $\Delta d$ is the distance between neighboring nonspecific sites, namely 1 bp. The definitions and dimensions of the quantities and parameters are listed in Table 1 in SI. The net flows of three components are generally described using the following equations:

3 $$\begin{aligned} \frac{\partial n_{\mathrm{NS}}(x, t)}{\partial t}= & {} D \frac{\partial ^2 n_{\mathrm{NS}}(x, t)}{\partial x^2} - k_{-}^{\mathrm{F-NS}} n_{\mathrm{NS}} (x,t) + k_{+}^{\mathrm{F-NS}} n_{\mathrm{F}}^{\mathrm{prt}} n_{\mathrm{F}}^{\mathrm{DNA}}(x, t) - J(t) \delta (x-x_{\mathrm{S}}), \end{aligned}$$

4 $$\begin{aligned} \frac{dn_{\mathrm{S}}(t)}{dt}= & {} -k_{-}^{\mathrm{F-S}} n_{\mathrm{S}} (t) + k_{+}^{\mathrm{F-S}} n_{\mathrm{F}}^{\mathrm{prt}} n_{\mathrm{F}}^{\mathrm{DNA}} (x_{\mathrm{S}},t) + J(t), \end{aligned}$$

5 $$\begin{aligned} J(t)= & {} k_{+}^{\mathrm{S-NS}} n_{\mathrm{NS}} (x_{\mathrm{S}},t) - k_{-}^{\mathrm{S-NS}} n_{\mathrm{S}} (t). \end{aligned}$$

Here, *D* is the diffusion coefficient of the protein along DNA, and the rate constants have a superscript showing the reaction pathway (F–S, F–NS, or S–NS) and subscript expressing forward reaction (+), or backward reaction (${-}$). Note that the dimensions (as physical units) of rate constants are defined by taking into account the difference of the dimensions between $n_{\mathrm{NS}} (x,t)$ and the other quantities $n_{\mathrm{S}}(t)$, $n_{\mathrm{F}}^{\mathrm{DNA}} (x,t)$ and so on. Assuming that the DNA length is finite and detailed balance holds between the isomerization of specific and nonspecific complexes at the specific site [$J(t) = k_{+}^{\mathrm{S-NS}} n_{\mathrm{NS}} (x_{\mathrm{S}},t) - k_{-}^{\mathrm{S-NS}} n_{\mathrm{S}} (t) = 0$ in Eqs. (3) and (4)], the dissociation constant of protein was calculated at some timescale slower than that of protein–DNA binding as follows:

6 $$\begin{aligned} K_{\mathrm{d}} = \frac{k_{-}^{\mathrm{F-S}}}{k_{+}^{\mathrm{F-S}}}. \end{aligned}$$

Since both $k_{+}^{\mathrm{F-S}}$ and $k_{-}^{\mathrm{F-S}}$ are independent of DNA length, $K_{\mathrm{d}}$, and thus the protein affinity for the specific site, is also independent. The analysis done in the discussion of Eq. (S11) in SI shows that detailed balance still holds at equilibrium irrespective of the addition of a diffusion term for finite DNA length. One may interpret that as DNA length increases, the number of forward pathways of TrpR from bulk to the specific site via nonspecific sites increases, enhancing the association to the specific site. Also, that of backward pathways increases, promoting the dissociation from the specific site. Such cancellation results in no dependence of DNA length on the affinity of TrpR. This conclusion is considered to be free from our choice of the one-dimensional diffusion/binding model.

**Figure 2:**
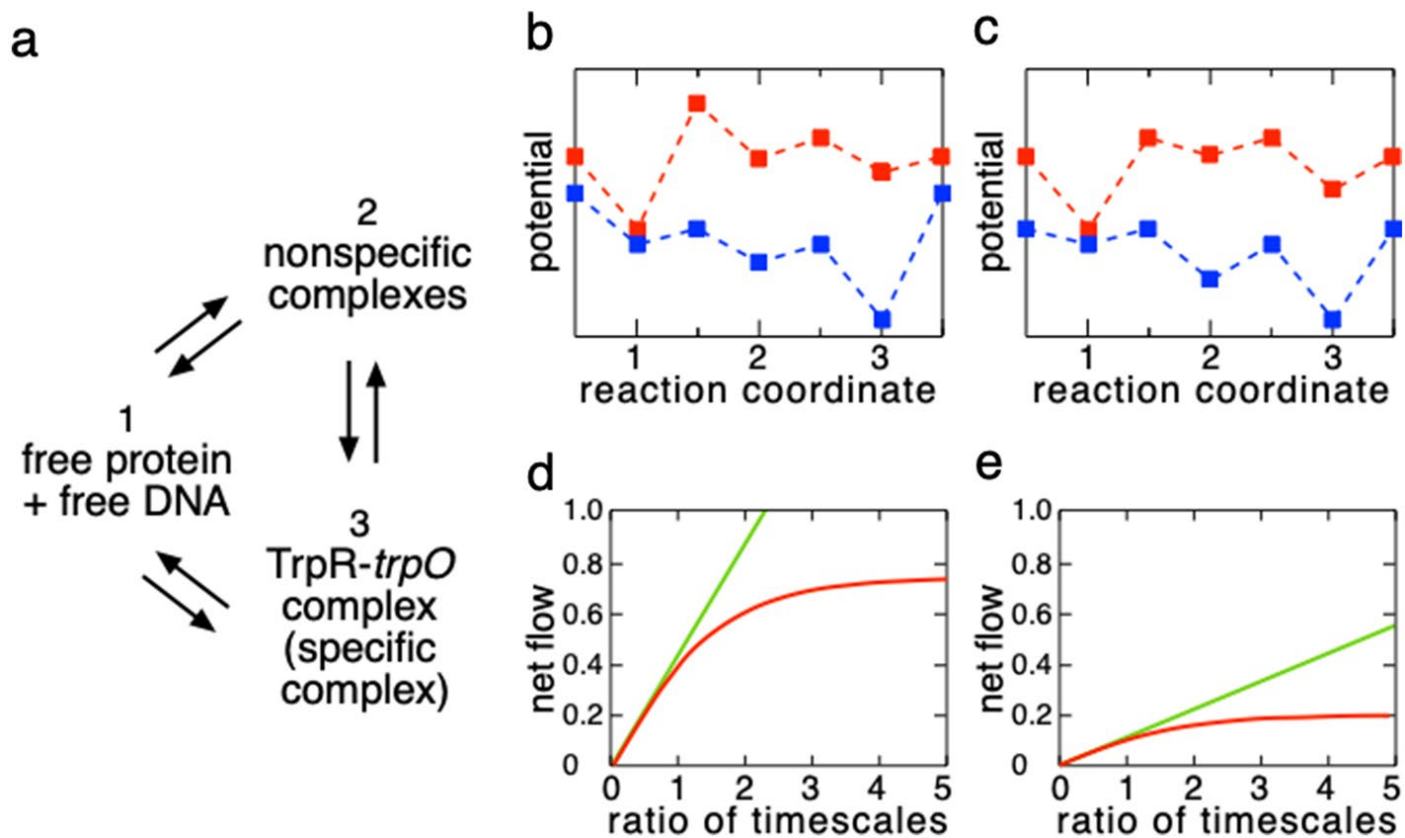
A chemical ratchet model to generate a net flow. (**a**) a network of chemical reactions among three states. The states 1, 2 and 3 correspond to dissociated protein/DNA, nonspecific complexes, and specific complex, respectively. (**b**) and (**c**) two different ratchets for this network, and each of them involves two alternating potentials A (blue) and B (red). The vertical and horizontal axes denote the potential and “reaction coordinate” i.e., succession of local minima and saddles on potentials, respectively. The local minima (the states at 1, 2, and 3) and the potential saddles are indicated by filled squares. The saddle on the left of the state 1 and that on the right of the state 3 are the same. (**d**) and (**e**) net flows caused by the ratchets corresponding to (**b**), and (**c**), respectively. The vertical and horizontal axes denote the corresponding net flow *J* caused by the ratchet, and the ratio of the timescales $t_{\mathrm{int}}{/}\max \left( t_A,t_B\right)$, respectively. The red line indicates the net flow *J*, and the green one does that caused by the matrix which is the time average of A and B [see SI Eq. (S36) for more detail].

### Chemical ratchet results in a deviation from detailed balance

Here we propose a working hypothesis to make the system deviate from detailed balance for some timescale. The key assumption of detailed balance is that the system attains a stationary distribution having time reversal symmetry. However, this assumption may not necessarily hold in all timescales in systems composed of modes with diverse timescales (unless canonical distribution would be prepared *a priori* as an ensemble). Even after fast degrees of freedom for protein/DNA system attain their equilibria, slower degrees of freedom may not, leading to deviation from detailed balance in the timescale of slow degree(s) of freedom^17^. Because deviation from detailed balance accompanies chemical changes in the system, we term this hypothesis as a ‘chemical ratchet.’

**Figure 3:**
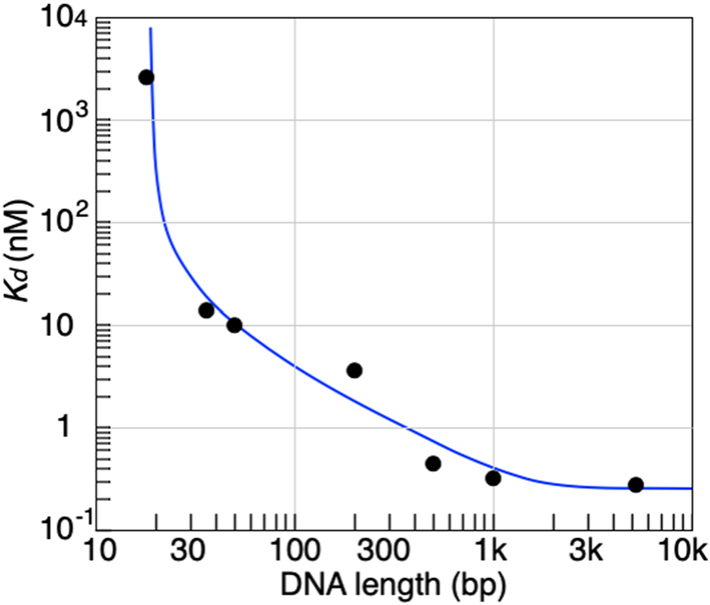
Antenna effect of TrpR. The filled circles denote $K_{\mathrm{d}}$ ($= \beta$, see the text) values experimentally obtained. The blue solid line is obtained for the best fit to Eq. (11), giving the following best-fitted values: $m_0 = 18 \, \hbox {bp}$, $d_0 = 625 \, \hbox {bp}$, $m_1 = 0.25$, and $m_2 = 1 \times 10^{-4}$.

Here we consider a simple chemical ratchet model switching between two potentials of mean force at every time interval $t_{\mathrm{int}}$. As shown in Fig. 2a, the system has three states 1, 2, and 3 corresponding to dissociated protein/DNA, nonspecific complexes, and specific complex, respectively. The ratchet switches between two different potentials A and B as in Fig. 2b,c. Let us suppose that on each potential the system satisfies the detailed balance between the three states. The timescales to reach equilibria on potentials A and B are denoted by $t_A$ and $t_B$, respectively. On the potential A shown in blue, state 3, which is the specific complex, is the most stable, and the flow is directed from state 1 to state 3 through state 2, i.e., from dissociated protein/DNA to specific complex via nonspecific complexes. On the potential B shown in red, the state 1, which is dissociated protein/DNA, becomes most stable. Therefore, the flow is directly from the state 3 to 1, i.e., dissociation from specific complex. This means that when the system switches alternatively between the two potentials for a timescale much slower than the timescales of equilibration on the potentials A and B, $t_A,t_B$, nonzero net flow occurs among the three kinetic states 1-3. In turn, when the system switches on a timescale much faster than $t_A$ and/or $t_B$, the system experiences only the averaged potential of the two, resulting in no net flow (Fig. 2d,e at the limit of $t_{\mathrm{int}}/\max \left( t_A,t_B\right) \rightarrow 0$). The above are two approximate explanations relevant only for extreme cases in terms of the ratio. Then, we would like to ask what will happen when the ratio is in an intermediate value.

Let us formulate this situation in the framework of master equations (see SI Eq. (S23)). The transition matrix *A* is, for example, defined as follows: $A_{i,j} = \exp \left( -\left( E_{i,j}^{(\mathrm A)}-E_{j}^{(\mathrm A)}\right) /T\right)$ for $i \ne j$, where $E_i^{(\mathrm A)}$ and $E_{i,j}^{(\mathrm A)}$ denote the potential of the *i*-th state, and the potential of the saddle separating the *i*-th and the *j*-th states on the potential A, respectively, with the Boltzmann constant set to 1 (the matrix *B* is also defined likewise for potential B). Suppose that the reactions described by transition matrices *A* and *B* alternate every time interval of timescale $t_{\mathrm{int}}$. Then, the net flow from the *j*-th to the *i*-th states $(j\ne i)$ of the ratchet is given by

7 $$\begin{aligned} J_{j \rightarrow i}=\int _{t_{\mathrm{int}}}^{2t_{\mathrm{int}}}\left( B_{i,j}P_j(t)-B_{j,i}P_i(t)\right) dt +\int _{0}^{t_{\mathrm{int}}}\left( A_{i,j}P_j(t)-A_{j,i}P_i(t)\right) dt, \end{aligned}$$

where $P_i(t)$ denotes the distribution of the *i*-th state at time *t*. To test our ratchet model, we calculate the time course of the net flow for two different potential landscapes (Fig. 2d,e). The net flow increases almost linearly for the ratio $t_{\mathrm{int}}/\max \left( t_A,t_B\right)$ being smaller than 1. On the other hand, the increase of the net flow *J* starts to deviate from linearity and saturates as the ratio exceeds 1.

The idea that the chemical ratchet induces deviation from detailed balance is applicable to any set of potentials. However, the amount of nonzero net flow that is generated depends on the timescale of switching and the topography of those potentials. For example, comparing the potentials B between Fig. 2b,c, we notice that the potential barrier of the saddle from state 2 to state 1 is larger in (b) than that in (c). Thus, more flow is generated from state 2 to state 3 for the ratchet in (b) than in (c), resulting in a larger nonzero net flow as shown in Fig. 2d,e. Moreover, the steeper the initial increase of the net flow is, the larger the saturated net flow is. This initial linear increase can be well reproduced by the time average of the matrices *A* and *B*, as shown by green lines in Fig. 2d,e (see SI Eq. (S36)). This implies that the average matrix provides us with a good approximation of the ratchet for $t_{\mathrm{int}}\lesssim \max (t_A,t_B)$.

Thus, the master equation is useful for a general description of the chemical ratchet. In the following, we present our model to explain the experimental results utilizing the rate equation rather than the master equation. The reason is that the rate equation is more suitable to take into account the specific mechanism involved in the reaction. The correspondence between the master equation and the rate equation is discussed in the reference^17^. Moreover, for a relevant model of the DNA/protein system, we only need to pay attention to a model corresponding to the matrix which is constant in time, without specifying the alternating matrices *A* and *B* [see SI Eq. (S36)]. In order to generate nonzero net flow permanently using the chemical ratchet, some external sources must exist to keep the slow degree(s) of freedom in non-equilibrium. In our experiments, however, we do not have to keep non-equilibrium conditions forever. It only suffices that non-equilibrium conditions are kept long enough so that nonzero net flow is observed in the experiment. As we will point out in Discussion, the timescales of slow degrees of freedom can be much longer than that of the experiment. Thus, we extend the concept of the ratchet to non-equilibrium steady conditions maintained by slow degrees of freedom. Note that the mechanism of our model is the same as the ratchets proposed by e.g.,^14,15^ in the sense that fluctuation of potentials gives rise to directional flow. Therefore, we call our model a “chemical ratchet” to highlight the point that it is an extension of those ratchets which operate under external energy sources. The number of cycles our ratchet performs during the experiment depends on the ratio of the two timescales: one for the experiment and the other for slow degrees of freedom to cause switching of the potentials.

### One-dimensional diffusion-binding model for protein–DNA system explains antenna effect observed in TrpR-***trpO*** system

How can one incorporate the concept of chemical ratchet in modeling TrpR–DNA system in which a $10^4$-fold antenna effect was observed in the affinity to *trpO* operator? Direct incorporation of chemical ratchet may require us to model at least two potentials of mean force among these three kinetic states, free TrpR and DNA, nonspecific complexes, and specific complex, which should be dependent on value(s) of the presumptive slow degree(s) of freedom buried in TrpR–DNA system. Instead, in this paper, we indirectly incorporate the concept of chemical ratchet into the framework of the one-dimensional diffusion/binding model. As discussed above [see SI Eq. (S11)], when detailed balance holds between the specific complex and the nonspecific complexes near the specific site, it was found that the dissociation constant of protein–DNA system cannot show any antenna effect at the timescale of the experiments. This observation suggests that some deviation from detailed balance is required primarily at the specific site in order to understand the antenna effect. Under stationary condition, the flux *J*(*t*) (Eq. 5) becomes constant, denoted by $J_0$ which is represented as $J_0 = \left( D d n_{\mathrm{NS}} (x)/dx | _{0+} \right) -\left( D d n_{\mathrm{NS}} (x) /d x | _{0-} \right)$, obtained by integrating the left and right hand sides of Eq. (3) from $x=0-$ to $x=0+$ with respect to *x*. While we assume the value of $J_0$ is nonzero, it is difficult to postulate the value of $J_0$, and hence we will determine the corresponding value (including its sign) so as to be consistent with the antenna effect observed in TrpR–DNA system. Here, the protein concentration in the bulk is set in enough excess over the DNA concentration for $n_{\mathrm{F}}^{\mathrm{prt}} \cong n_{\mathrm{tot}}^{\mathrm{prt}}$ to hold. In addition, we introduce new approximations $n_{\mathrm{F}}^{\mathrm{DNA}}(x,t) \cong n_{\mathrm{tot}}^{\mathrm{DNA}} \; (x \ne x_{\mathrm{S}})$ and $n_{\mathrm{F}}^{\mathrm{DNA}}(x_{\mathrm{S}},t) \cong n_{\mathrm{tot}}^{\mathrm{DNA}} - n_{\mathrm{S}}(t)$, which are rationalized in the SI after Eqs. (S1–S3), to eliminate some variables as

8 $$\begin{aligned} \frac{\partial n_{\mathrm{NS}}(x,t)}{\partial t}= & {} D \frac{\partial ^2 n_{\mathrm{NS}}(x,t)}{\partial x^2} - k_{-}^{\mathrm{F-NS}} n_{\mathrm{NS}}(x,t) + k_{+}^{\mathrm{F-NS}} n_{\mathrm{tot}}^{\mathrm{prt}} n_{\mathrm{tot}}^{\mathrm{DNA}} - J_0 \delta (x) = 0, \end{aligned}$$

9 $$\begin{aligned} \frac{d n_{\mathrm{S}}(t)}{d t}= & {} - (k_{-}^{\mathrm{F-S}} + k_{+}^{\mathrm{F-S}}n_{\mathrm{tot}}^{\mathrm{prt}}) n_{\mathrm{S}}(t) + k_{+}^{\mathrm{F-S}}n_{\mathrm{tot}}^{\mathrm{prt}}n_{\mathrm{tot}}^{\mathrm{DNA}} + J_0 = 0, \end{aligned}$$

where the specific site locates at $x = 0$, and $\delta (x)$ denotes Dirac’s delta function. $J_0$ corresponds to Eq. (5) at $x = 0$, and positive (negative) $J_0$ expresses incoming (outgoing) diffusion flow into (from) the specific site from (to) nonspecific parts. The flow $J_0$ is found to be represented by $\displaystyle J_0=\frac{2 D n_0}{d_0} \tanh \frac{l}{d_0}$ [see SI Eqs. (S16) and (S18)]. In brief, $J_0$ arises from $\sim \,D \displaystyle { \frac{\partial }{\partial x} n_{\mathrm{NS}}(x) \sim D \frac{n_0}{d_0}}$ where $n_0$ is some constant having the same dimension as $n_{\mathrm{NS}}(x)$. *l* is the length of nonspecific DNA segment from *trpO* to an end. $d_0$ denotes the average length of DNA covered with protein diffusion without dissociation from DNA, and equals to Eq. $\sqrt{D/k_{\mathrm{-}}^{\mathrm{F-NS}}}$. The term $\tanh (l/d_0)$ in $J_0$ represents the following: for instance, when TrpR associates with any of nonspecific parts on the DNA and diffuses into the specific site, TrpR may leave from the nonspecific parts before it finds the specific site. That is, for the total length of nonspecific parts *l*, the diffusion flow along the DNA should be “saturated” for $l > d_0$, because any region more distant than $d_0$ gives no contribution to flow. Note that, while non-vanishing $J_0$ makes the system deviate from detailed balance, the value of $J_0$ will be determined so as to be consistent with the observed antenna effect of TrpR–DNA system.

By solving for $n_{\mathrm{S}}$ using Eq. (9) and inserting $n_{\mathrm{S}}$, $n_{\mathrm{F}}^{\mathrm{prt}}$ and $n_{\mathrm{F}}^{\mathrm{DNA}}(0)$ corresponding to $[\hbox {TrpR-}trpO \; \hbox {complex}]$, $[\hbox {TrpR}]$, and $[\hbox {free} \; trpO]$ into the definition of $\beta$ in Eq. (2) [see the discussion after Eq. (S21) in SI], we obtain Eq. (10),

10 $$\begin{aligned} \beta = \displaystyle \frac{n_{\mathrm{F}}^{\mathrm{prt}} n_{\mathrm{F}}^{\mathrm{DNA}}(0)}{n_{\mathrm{S}}} \cong \frac{k_{-}^{\mathrm{F-S}} n_{\mathrm{tot}}^{\mathrm{prt}} n_{\mathrm{tot}}^{\mathrm{DNA}}}{k_{+}^{\mathrm{F-S}}n_{\mathrm{tot}}^{\mathrm{prt}} n_{\mathrm{tot}}^{\mathrm{DNA}} + J_0} = \frac{k_{-}^{\mathrm{F-S}} n_{\mathrm{tot}}^{\mathrm{prt}} n_{\mathrm{tot}}^{\mathrm{DNA}}}{ k_{+}^{\mathrm{F-S}} n_{\mathrm{tot}}^{\mathrm{prt}} n_{\mathrm{tot}}^{\mathrm{DNA}} + \displaystyle \frac{2 D n_0}{d_0} \tanh \frac{l}{d_0} }. \end{aligned}$$

Equation (10) gives an expression of $\beta$ (usually referred at equilibrium) under the existence of nonzero diffusion flow. Recalling the experimental result that $\beta$ monotonically decreases about $10^4$-fold as the DNA length increases, this means that $n_0$ is positive (i.e., $J_0>0$), since all the other parameters are positive in Eq. (10). Positive $J_0$ implies that $n_S$ involves the *incoming* flow of TrpR to the specific site along DNA, $J_0(l)/k_{-}^{\mathrm{F-S}}$, in addition to the term defined at equilibrium, $k_{+}^{\mathrm{F-S}} n_{\mathrm{tot}}^{\mathrm{prt}} n_{\mathrm{tot}}^{\mathrm{DNA}} / k_{-}^{\mathrm{F-S}}$.

For fitting of the experimental result, we rewrite Eq. (10) as

11 $$\begin{aligned} \beta = \frac{m_1}{m_2 + \tanh \left( \displaystyle \frac{\mathrm{DNA \; length} - m_0}{2 d_0} \right) }. \end{aligned}$$

Note that we take ($2l + m_0$) as the DNA length where $m_0$ represents the finite size of specific site implicitly and the factor 2 is due to our experimental design where the length of nonspecific parts are equal for each side from the specific site. We determined $m_0$, $m_1$, $m_2$, and $d_0$ to be best fitted to Fig. 3 where $\log \beta$ is plotted against log(DNA length). A satisfactory fitting is obtained for four order of magnitude of $\beta$. The constant $\beta$ could be considered as the dissociation constant $K_{\mathrm{d}}$ extended in the non-equilibrium steady state. Note also that, when $J_0$ vanishes, Eq. (10) turns to the definition of dissociation constant $\displaystyle K_{\mathrm{d}} = \frac{k_{-}^{\mathrm{F-S}}}{k_{+}^{\mathrm{F-S}}}$ at equilibrium. The errors were more sensitive to the values of $m_0$ and $d_0$ (compared to those of $m_1$ and $m_2$) whose best fitted values 18 bp and 625 bp were consistent with the previous studies^24,25^. While the solid identification of the actual value of $J_0$ is limited only for seven experimental data points, the positive $J_0$ reflects that the underlying TrpR and DNA binding interaction energy at the specific site is greater than those at nonspecific sites, and enables protein of complexes at *trpO* to dissociate from DNA, but not to transfer into the neighboring nonspecific parts by one-dimensional diffusion, introducing asymmetry in association and dissociation.

## Discussion

In this simplified model the deviation is expressed to exist permanently, but it should be noted that the deviation from detailed balance should take place at least for the timescale of TrpR–DNA binding. In biomolecules, there exist multiple timescales for various degrees of freedom across many different orders to reach equilibrium. The timescale of DNA–protein binding is usually of the order of milliseconds to seconds, while that of protein-conformational changes can be slower: about a minute for *Neurospora* glutamate dehydrogenase^26^ as well as rat liver glucokinase^27^, an hour for Vaccinia topoisomerase I^28^, and a year or longer for prion proteins^29^. Thus, even when some degrees of freedom have attained equilibrium, the others may not necessarily reach equilibrium. This implies that deviation from detailed balance does not necessarily hold permanently in practice when there exist some degrees of freedom that are slower than that of the observed antenna effects. The deviation from detailed balance is thus sufficient to hold only in the timescale of TrpR–DNA binding. As long as those slow degrees of freedom remain in non-equilibrium, the chemical ratchet can be one possible scenario to explain the antenna effects. We admit that there still remains the very fundamental question of what kinds of slow degree(s) of freedom can cause deviation from detailed balance in the TrpR–DNA experiment. In this section, we address some possible scenarios.

DNA bending can affect protein binding to the bent DNA. Some proteins are known to form a specific complex with a bent DNA^30^. For example, *E. coli* integration host factor (IHF) binds to the specific site and bends DNA at an angle larger than $160^\circ$, which might prevent the protein from sliding out from the specific site. A time-resolved fluorescence resonance energy transfer (FRET) analysis of DNA bending in the presence of IHF showed that thermally induced DNA bending merely triggers its DNA binding, and the DNA in a complex is largely bent at the cost of the stabilization energy of specific interaction^31^. Thus, a large change in DNA conformation in a complex can coupled with the formation of specific interaction in structural and energetic manner, suggesting that a large DNA bending in a complex can drastically change the pathway and the velocity of its dissociation.

Bacterial repressors including TrpR are known to insert their helix-turn-helix motifs in the DNA groove to form their specific complexes^30,32^. A large bending may open the DNA groove to induce dissociation of the bound protein. The large DNA bending is expected to break many interactions between protein and DNA in the specific complex, which decreases the affinity of the protein at specific site. When the protein tracks the DNA groove during its one-dimensional diffusion, the bent groove can induce dissociation, because the specific interactions are maintained with Angstrom accuracy^30,33^. Moreover, a DNA bent is known to inhibit one-dimensional diffusion^34^. These results are consistent with a chemical ratchet with a slow switch of DNA bent in the complex. Note that small DNA bending can be thermally induced frequently with timescale only at several microseconds^35^, which is much shorter than the typical timescale of DNA–protein binding. DNA bending postulated here is different from those and supposed to be much less frequent and large enough to break many stable interactions between protein and DNA in the specific complex to result in a decrease of the affinity of the protein at specific site.

Another possible source of slow degrees of freedom might be conformational change of the protein. A protein in the specific site may alter the stable complex conformation to an unstable one so as to trigger the system switch. There are no reports for detecting multiple conformations and the conformational change in TrpR. However, single-molecule measurements revealed that several DNA-binding proteins have multiple diffusion coefficients for sliding along DNA^36–38^. It suggests the presence of at least two conformations with different sliding ability. The conformational change of proteins bound to DNA would occur at some timescale longer than that of the binding reaction speculated to be several tens of milliseconds, which satisfies the timescale required for chemical ratchet. Accordingly, it is not surprising even if TrpR shows such slow conformational change.

In summary, the (reaction) system can deviate from detailed balance when some degree(s) of freedom slower than the timescale of interest exist(s), in this paper, the timescale of TrpR–DNA binding. This holds even when the system resides under thermal bath, unless a canonical ensemble would be pre-prepared. This can occur without an apparent source such as temperature gradient. Then, the ratchet keeps operating as far as those degrees of freedom with longer timescales remain to deviate from detailed balance. Moreover, analyzing the mechanism of ratchets may be inevitable for understanding reaction processes in biological systems.

## Materials and methods

### Protein and DNA

*E. coli* TrpR protein was provided by Dr. Jannette Carey. All the DNA fragments contained only the intact chromosomal DNA near the *trpO* site of *trpR* gene^25^, except the 5.2 kbp and 18 bp ones. The 5.2 kbp fragment included a 2.1 kbp vector sequence of pRPG16 in each end, and the 18 bp one was a hairpin duplex with a loop of five cytidine residues to prevent dissociation of the short stem duplex. The fragments shorter than 500 bp were purified by electrophoresis in an 8% polyacrylamide gel, followed by simple diffusion from the crushed gel slices. The longer fragments were purified by electrophoresis in a 0.7% agarose gel and recovered using the Rapid Gel Extraction kit (Life Technologies).

### Hydroxyl radical footprinting

All the binding experiments were performed in 10 mM sodium-phosphate buffer (pH 6.5) containing 25 mM NaCl, with or without 0.25 mM L-tryptophan as the corepressor. To obtain footprints of TrpR protein on DNA, the 5’-end of the hairpin DNA was labeled with [$\gamma$-$^{32}$P]ATP (6000 Ci/mol, NEN) using T4 polynucleotide kinase (Takara, Kyoto). The unreacted ATP was removed by passage through a SpinColumn G-50 (Pharmacia). For DNA fragments shorter than 500 bp, the 5’-end of the primer was similarly labeled before synthesis of the fragment by PCR. For longer fragments, a primer corresponding to the sequence 100 bp upstream of the *trpO* site was labeled, and then extended by Sequenase (Stratagene) using the footprinted DNA as a template. The cleavage reaction was performed for 3 m in the presence of $2.5 \, \mu {\hbox {M Fe}}^{2+}$-EDTA mix^22^, 0.25 mM sodium ascorbate, and 0.015% hydrogen peroxide at room temperature. The concentrations of DNA were 20 nM, 2 nM, 1 nM, 1 nM, and 10 pM for the hairpin DNA, 36 bp, 50 bp, 200 bp, and the longer ones, respectively. To satisfy the single-cutting condition, we stopped the cleavage by an addition of glycerol to 20% when 20% of the full-length DNA fragment had disappeared. The radioactivity was measured by a phosphoimager BAS1500 (Fuji Film, Tokyo) and normalized within individual lanes for correction of loading errors. The band density in the absence of TrpR was subtracted as the background to calculate the enhanced densities. Fitting the data to Eq. (1) was carried out by the least-squares method with MacCurveFit 1.5.

## Supplementary information

Supplementary material 1
