# Supplementary material for: Dependence of DNA length on binding affinity between TrpR and trpO of DNA
Source: Sci Rep. 2020 Sep 24;10:15624. doi: 10.1038/s41598-020-71598-3 (PMC7518442; doi:10.1038/s41598-020-71598-3)
Supplement: Supplementary file 1 — Supplementary material 1 [file 41598_2020_71598_MOESM1_ESM.pdf]

Supplementary

Dependence of DNA length on binding affinity between TrpR and *trpO* of DNA

Nobuo Shimamoto, Mikito Toda, Shigetoshi Nara, Tamiki Komatsuzaki, Kiyoto Kamagata, Takashi Kinebuchi, Jun-ichi Tomizawa

In this supplementary section, we describe rather detailed mathematical derivation about the important formulae in the paper.

A Reaction-Diffusion Equation

We rewrite a set of model equations to treat the present problem introduced in the paper, Eqs. (3) $\sim$ (5).

$$\frac{\partial n_{\mathrm{NS}}(x,t)}{\partial t} = D\frac{\partial^2 n_{\mathrm{NS}}(x,t)}{\partial x^2} - k_-^{\mathrm{F-NS}} n_{\mathrm{NS}}(x,t) + k_+^{\mathrm{F-NS}} n_{\mathrm{F}}^{\mathrm{prt}} n_{\mathrm{F}}^{\mathrm{DNA}}(x,t) - J(t)\delta(x), \tag{S1}$$

$$\frac{dn_{\mathrm{S}}(t)}{dt} = -k_-^{\mathrm{F-S}} n_{\mathrm{S}}(t) + k_+^{\mathrm{F-S}} n_{\mathrm{F}}^{\mathrm{prt}} n_{\mathrm{F}}^{\mathrm{DNA}}(0,t) + J(t), \tag{S2}$$

$$J(t) = k_+^{\mathrm{S-NS}} n_{\mathrm{NS}}(0,t) - k_-^{\mathrm{S-NS}} n_{\mathrm{S}}(t), \tag{S3}$$

where the specific site is set to be located at the origin, $x_{\mathrm{S}} = 0$, without loss of generality and the definitions of all variables, parameters, and constants are given in the paper. As shown in Eq. (S3), we suppose that the specific complex is formed by isomerization of a nonspecific complex at the specific site, because *trpO* is stochastically overlooked during one-dimensional diffusion across the site.

The conservation of each DNA site is $n_{\mathrm{F}}^{\mathrm{DNA}}(x,t) = n_{\mathrm{tot}}^{\mathrm{DNA}} - n_{\mathrm{NS}}(x,t)\Delta d$ for $x \neq x_{\mathrm{S}}$ or $n_{\mathrm{F}}^{\mathrm{DNA}}(x_{\mathrm{S}},t) = n_{\mathrm{tot}}^{\mathrm{DNA}} - n_{\mathrm{S}}(t) - n_{\mathrm{NS}}(x_{\mathrm{S}},t)\Delta d$. In the following, we omit $n_{\mathrm{NS}}(x_{\mathrm{S}},t)\Delta d$ as an approximation under our experimental conditions as rationalized after Eq. (S21). This omission is consistent to the introduction of the diffusion term in Eq. (S1) because the expression is for the diffusion on naked DNA with no collision between protein molecules. The omission simplifies the equations a great deal, and Eq. (3) and Eq. (4) in the text are respectively converted into Eq. (8) and (9).

Stationary Solution under finite DNA-length condition

As described in Eq. (8) of the paper, let us consider stationary solutions of these kinetic reaction-diffusion equations by setting $\partial n_{\mathrm{NS}}/\partial t = 0$ and $dn_{\mathrm{S}}/dt = 0$ in Eqs. (S1) and (S2), respectively.

Under stationary conditions, the flux $J(t)$ expressed by Eq. (S3) is put to be constant, denoted by J_0, which is obtained by integrating the left and right hand sides of Eq. (S1) from $x = 0-$ to $x = 0+$ with respect to x:

$$J_0 = \left(D\frac{dn_{\mathrm{NS}}(x)}{dx}\bigg|_{0+} \right) - \left(D\frac{dn_{\mathrm{NS}}(x)}{dx}\bigg|_{0-} \right). \tag{S4}$$

Eq. (S1) is rewritten into a simpler form as

$$\frac{d^2 n_{\mathrm{NS}}(x)}{dx^2} - \frac{1}{d_0^2} n_{\mathrm{NS}}(x) = -g_0^2 + q_0\delta(x), \tag{S5}$$

where $n_{\mathrm{F}}^{\mathrm{prt}} n_{\mathrm{F}}^{\mathrm{DNA}}(x,t)$ in Eq. (S1) is replaced by $n_{\mathrm{tot}}^{\mathrm{prt}} n_{\mathrm{tot}}^{\mathrm{DNA}} =$ const. as described above, and

$$d_0 = \sqrt{\frac{D}{k_-^{\mathrm{F-NS}}}}, \qquad g_0^2 = \frac{k_+^{\mathrm{F-NS}}}{D} n_{\mathrm{tot}}^{\mathrm{prt}} n_{\mathrm{tot}}^{\mathrm{DNA}}, \qquad q_0 = \frac{J_0}{D}. \tag{S6}$$

Here, d_0 denotes the average length of DNA that is covered with proteins diffusing without dissociating from DNA. Similarly, under stationary conditions, Eq. (S2) is rewritten as

$$-k_-^{\mathrm{F-S}} n_{\mathrm{S}} + k_+^{\mathrm{F-S}} n_{\mathrm{F}}^{\mathrm{prt}} n_{\mathrm{F}}^{\mathrm{DNA}}(0) + J_0 = 0, \tag{S7}$$

where $n_{\mathrm{F}}^{\mathrm{DNA}}(0)$ denotes $n_{\mathrm{F}}^{\mathrm{DNA}}(x=0)$, i.e., the concentration of DNA with its specific site empty. Furthermore, let us impose the following boundary conditions at the edge of DNA chain (namely, a finite length $2l$ of DNA) as

$$\frac{d}{dx}n_{\mathrm{NS}}(x)\bigg|_{x=l}=0 \quad \text{and} \quad \frac{d}{dx}n_{\mathrm{NS}}(x)\bigg|_{x=-l}=0, \tag{S8}$$

which means that there is no escaping (diffusion) flow of protein from the edges of DNA ($x=\pm l$) because diffusion flow is represented, in general, as $-D(dn(x)/dx)$, where D is diffusion constant and $n(x)$ is concentration of substance concerned.

Case 1: $J_0=0$

When detailed balance holds between the specific complex and the nonspecific complex near the specific site ($J_0=0$ in Eq. (S5) and (S7)), then the equation to solve is

$$\frac{d^2 n_{\mathrm{NS}}(x)}{dx^2}-\frac{1}{d_0^2}n_{\mathrm{NS}}(x)=-g_0^2, \qquad \text{and} \qquad -k_-^{\mathrm{F-S}}n_{\mathrm{S}}+k_+^{\mathrm{F-S}}n_{\mathrm{F}}^{\mathrm{prt}}n_{\mathrm{F}}^{\mathrm{DNA}}(0)=0. \tag{S9}$$

The former is non-homogeneous, second-order, ordinary differential equation. The solution is represented as the summation of the general solution for the homogeneous second order ordinary differential equation of $g_0^2=0$ and a special solution of this equation. The special solution $n_{\mathrm{NS}}^{\mathrm{special}}$ is determined as $n_{\mathrm{NS}}^{\mathrm{special}}=d_0^2g_0^2=$ const., and, hence the solution is

$$n_{\mathrm{NS}}(x)=A\cosh\left(\frac{x}{d_0}\right)+B\sinh\left(\frac{x}{d_0}\right)+d_0^2g_0^2, \tag{S10}$$

where A and B are constant real numbers and determined so as to satisfy the boundary condition, Eq. (S8). The boundary condition $\frac{d}{dx}n_{\mathrm{NS}}(x)\Big|_{x=\pm l}=0$ is only satisfied with $A=B=0$. Thus finally, the solution is $n_{\mathrm{NS}}(x)=d_0^2g_0^2=\frac{k_+^{\mathrm{F-NS}}n_{\mathrm{tot}}^{\mathrm{prt}}n_{\mathrm{tot}}^{\mathrm{DNA}}}{k_-^{\mathrm{F-NS}}}=$ const., and $n_{\mathrm{NS}}(x)$ results in spatial homogeneity and no diffusion flow exists. It tells that detailed balance is maintained at all non-specific sites even with including of additional diffusion terms to kinetic equation, i.e., under the stationary condition where $dn_{\mathrm{NS}}(x,t)/dt=dn_{\mathrm{S}}/dt=0$ with $J_0=0$, we obtain the conventional definition of the dissociation constant K_{d} as

$$-k_-^{\mathrm{F-S}}n_{\mathrm{S}}+k_+^{\mathrm{F-S}}n_{\mathrm{F}}^{\mathrm{prt}}n_{\mathrm{F}}^{\mathrm{DNA}}(0)=0 \qquad \therefore\ K_{\mathrm{d}}=\frac{n_{\mathrm{F}}^{\mathrm{prt}}n_{\mathrm{F}}^{\mathrm{DNA}}(0)}{n_{\mathrm{S}}}=\frac{k_-^{\mathrm{F-S}}}{k_+^{\mathrm{F-S}}} \tag{S11}$$

and K_{d} is independent of the DNA length. This implies that when the term J_0 permanently vanishes in Eq. (S5) $\sim$ (S7), i.e., detailed balance holds between the specific complex and the nonspecific complex near the specific site, the stationary solution, $n_{\mathrm{NS}}(x)$ is found to be spatially uniform, which contradicts the antenna effect observed experimentally.

Case 2: $J_0\neq 0$

The above consequence derived from $J_0=0$ suggests that some deviation from detailed balance at least for some timescale of TrpR-DNA binding is required at the specific site to capture the antenna effect. This implies that a solution of Eq. (S5) in the case of $J_0\neq 0$ may explain the experimental results. From Eq. (S4), $J_0\neq 0$ means that $n_{\mathrm{NS}}(x)$ is continuous but not differentiable at the specific site ($x=0$), that is, singular point in $n_{\mathrm{NS}}(x)$ because $\frac{dn_{\mathrm{NS}}(x)}{dx}\Big|_{0-}\neq\frac{dn_{\mathrm{NS}}(x)}{dx}\Big|_{0+}$.

Now, in solving Eq. (S5), we assume the same boundary conditions at the edge of DNA chain ($x=\pm l$) (see Eq. (S8)), and $J_0\neq 0$. The set of equations to be solved simultaneously becomes

$$\frac{d^2 n_{\mathrm{NS}}(x)}{dx^2}-\frac{1}{d_0^2}n_{\mathrm{NS}}(x)+g_0^2=q_0\delta(x), \qquad \frac{d}{dx}n_{\mathrm{NS}}(x)\bigg|_{x=\pm l}=0, \tag{S12}$$

under the condition that $n_{\mathrm{NS}}(x)$ is, at $x=0$, continuous but non-differentiable. Therefore, we should solve the above equation individually for $x>0$ and $x<0$ with a set of two undetermined constants, say, $A^{(+)},B^{(+)}$ and $A^{(-)},B^{(-)}$. Eq. (S10) gives us a possible candidate solution in the individual region, i.e.,

$$n_{\mathrm{NS}}(x)=A^{(\pm)}\cosh\left(\frac{x}{d_0}\right)+B^{(\pm)}\sinh\left(\frac{x}{d_0}\right)+d_0^2g_0^2, \tag{S13}$$

In $x>0$ region, when we impose the boundary condition $\frac{d}{dx}n_{\mathrm{NS}}(x)\Big|_{x=+l}=0$, then we can get

$$n_{\mathrm{NS}}(x)=A^{(+)}\left[-\cosh\left(\frac{x}{d_0}\right)+\tanh\left(\frac{l}{d_0}\right)\sinh\left(\frac{x}{d_0}\right)\right]+d_0^2g_0^2 \qquad (0<x\leq l). \tag{S14}$$

Executing the same procedure for $x < 0$ and unifying them under the condition that $n_{\rm NS}(x)$ is continuous at $x = 0$ (resulting in $A^{(+)} = A^{(-)}$), we obtain

$$n_{\rm NS}(x) = n_0 \left[-\cosh\left(\frac{|x|}{d_0}\right) + \tanh\left(\frac{l}{d_0}\right) \sinh\left(\frac{|x|}{d_0}\right) \right] + d_0^2 g_0^2, \tag{S15}$$

where n_0 $(= A^{(+)} = A^{(-)})$ is some constant having the same unit as $n_{\rm NS}(x,t)$. By substituting Eqs. (S14) into Eq. (S4), we can obtain

$$J_0 = \frac{2Dn_0}{d_0} \tanh \frac{l}{d_0}. \tag{S16}$$

In summary, the solution of Eq. (S12) is given by

$$n_{\rm NS}(x) = d_0^2 g_0^2 - n_0 \left[\cosh\frac{|x|}{d_0} - \tanh\frac{l}{d_0} \sinh\frac{|x|}{d_0} \right] \qquad (-l \le x \le l), \tag{S17}$$

where the diffusion flow and $d_0^2 g_0^2$ are represented as

$$J_0 = \frac{2Dn_0}{d_0} \tanh \frac{l}{d_0}, \qquad d_0^2 g_0^2 = \frac{k_+^{\rm F-NS} n_{\rm tot}^{\rm prt} n_{\rm tot}^{\rm DNA}}{k_-^{\rm F-NS}}. \tag{S18}$$

In the expression of $n_{\rm NS}(x)$, the first term represents a spatially-uniform term that is independent of x, which is also the solution of $n_{\rm NS}$ at $J_0 = 0$ where detailed balance holds. The second term corresponds to the consequence of deviation from detailed balance between the specific complex and the nonspecific complex near the specific site. One can see that, as the length from the specific site is much longer than a typical diffusion length (d_0) along DNA whose l is sufficiently larger than d_0, i.e., $|x| >> d_0$ and $\tanh(l/d_0) \sim 1$, the second term becomes negligibly small compared with the first term. This means that the concentration of nonspecific complex per unit length $n_{\rm NS}(x)$ located further from the specific site ($x = 0$) than d_0 is equivalent to that expected when detailed balance holds. In contrast, in the neighborhood of the specific site within d_0, the more $n_{\rm NS}(x)$ deviates from the equilibrium value, the closer the location of the nonspecific site is to the specific site. As described in the paper, J_0 expresses diffusion flow along DNA into the specific site, arising from $\sim D\frac{\partial}{\partial x} n_{\rm NS}(x) \sim D\frac{n_0}{d_0}$ where n_0 is some constant having the same dimension as $n_{\rm NS}(x)$. The term $\tanh(l/d_0)$ in J_0 represents that, compared to the diffusion length d_0 to the total length of nonspecific parts l, the diffusion flow along the DNA should be "saturated" for $l > d_0$, because any region more distant than d_0 gives no contribution to flow.

It is noted that J_0 represented by Eq. (S4) assumed to deviate from zero results in x-dependence for the nonspecific complex concentration $n_{\rm NS}$. However, the question of whether J_0 is positive or negative, moreover what value could be assigned for J_0 requires consistency with the observed experimental data. In the next subsection, we present a phenomenological theory of binding affinity depending on DNA length that enables us to answer this question.

A phenomenological theory of binding affinity depending on DNA length

The final problem is to calculate the quantity corresponding to "dissociation constant" whose concept could be valid under the condition that detailed balance holds entirely. The experimental values of $K_{\rm d}$ in the present work are calculated using Eq. (1) in the paper with setting the experimental condition "largely excessive amount of protein in solution".

The results manifest that the experimental values of $K_{\rm d}$ exhibits *significant* DNA-length dependence, which contradicts with the assumption of detailed balance that may usually be assumed to hold entirely over any timescale. Here the conventional expression of "dissociation constant" cannot, theoretically, explain the experimental observation. Nevertheless, in the present experiments, $K_{\rm d}$ calculated using Eq. (1) from the experimentally measured data of $n_{\rm S}$ shows the DNA-length dependence. It suggests that certain effective or apparent dissociation constant depending on DNA-length may have to exist as the form of

$$K_{\rm d}^{\rm experiment}(l) = \frac{n_{\rm F}^{\rm prt} n_{\rm F}^{\rm DNA}}{n_{\rm S}^{\rm experiment}(l)}, \tag{S19}$$

which is denoted by "β" in the paper.

From the approximated conservation of *trpO*, $n_{\rm F}^{\rm DNA}(0) \cong n_{\rm tot}^{\rm DNA} - n_{\rm S}$, Eq. (9) for the stationary state is given as

$$-(k_-^{\rm F-S} + k_+^{\rm F-S} n_{\rm tot}^{\rm prt}) n_{\rm S} + k_+^{\rm F-S} n_{\rm tot}^{\rm prt} n_{\rm tot}^{\rm DNA} + J_0 = 0. \tag{S20}$$

If the definition of K_d shown in Eq. (S11) is extended to this stationary state, we obtain

$$K_\mathrm{d}^\mathrm{theory} = \frac{n_\mathrm{F}^\mathrm{prt} n_\mathrm{F}^\mathrm{DNA}(0)}{n_\mathrm{S}} = n_\mathrm{F}^\mathrm{prt}\left(\frac{n_\mathrm{tot}^\mathrm{DNA}}{n_\mathrm{S}} - 1\right) \cong \frac{k_-^\mathrm{F-S} n_\mathrm{tot}^\mathrm{DNA} - J_0}{k_+^\mathrm{F-S} n_\mathrm{tot}^\mathrm{DNA} + \dfrac{J_0}{n_\mathrm{tot}^\mathrm{prt}}} = \frac{k_-^\mathrm{F-S} n_\mathrm{tot}^\mathrm{DNA} - \dfrac{2Dn_0}{d_0}\tanh\dfrac{l}{d_0}}{k_+^\mathrm{F-S} n_\mathrm{tot}^\mathrm{DNA} + \dfrac{2Dn_0}{d_0 n_\mathrm{tot}^\mathrm{prt}}\tanh\dfrac{l}{d_0}}, \tag{S21}$$

since we have assumed $n_\mathrm{F}^\mathrm{prt} \cong n_\mathrm{tot}^\mathrm{prt}$.

In deriving Eq. (S21), we have omitted the term $n_\mathrm{NS}(x,t)\Delta d$. The reason is as follows. In our experiment, the dependence of n_S on $n_\mathrm{tot}^\mathrm{prt}$ is the same as that of one-to-one binding which produces a single complex as shown in Fig. (1c). On the other hand, such results are not generally guaranteed for the binding reaction with multiple binding sites for specific and nonspecific complexes. Therefore, we have omitted the concentrations of nonspecific complex at *trpO* and other sites from the conservations of DNA sites. This omission is also based on the result shown in Fig. (1c), the curve for the simple one-to-one binding. If their concentrations were significant in our experimental conditions, or if these sites were significantly occupied with nonspecific complexes, the curve would significantly and systematically deviate from the observed one.

Moreover, in our experiment, we have defined β in the same way as K_d in Eq. (21), and have determined the value of β by changing $n_\mathrm{tot}^\mathrm{prt}$ at a fixed value of $n_\mathrm{tot}^\mathrm{DNA}$. Notably, we have obtained β as a constant which is independent of $n_\mathrm{tot}^\mathrm{prt}$. If β is represented by the extended K_d with J_0, this independence is consistent with the case when both of the following two conditions are satisfied: (i) n_0 is proportional to n_tot^{prt}, and (ii) $k_-^\mathrm{F-S} n_\mathrm{tot}^\mathrm{DNA} \gg \dfrac{2Dn_0}{d_0}\tanh\dfrac{l}{d_0}$. Then, we obtain the following expression

$$K_\mathrm{d}^\mathrm{theory} \cong \frac{k_-^\mathrm{F-S} n_\mathrm{tot}^\mathrm{DNA}}{k_+^\mathrm{F-S} n_\mathrm{tot}^\mathrm{DNA} + \dfrac{2Dn_0}{d_0 n_\mathrm{tot}^\mathrm{prt}}\tanh\dfrac{l}{d_0}}, \tag{S22}$$

which leads to Eq. (10) in the text.

Eq. (S22) should correspond to $K_\mathrm{d}^\mathrm{experiment}(l)$ under the existence of nonzero diffusion flow. As addressed in the paper, by considering the experimental fact on β described above, J_0 should be positive as expected from enhanced binding for a longer DNA. This phenomenological theory enables us to extend the concept of "association" and "affinity" to non-equilibrium stationary states.

Chemical ratchet

Relevance of detailed balance

Let us first revisit the concept of detailed balance and clarify the assumptions underlying its derivation, following the argument by van Kampen in Ch. V §6 of the reference[1]. The crucial assumptions for the derivation of detailed balance are the following.

(i) The microscopic process has time reversal symmetry. That is, any trajectory propagated backward in time also exists as a solution of the microscopic dynamical equation.

(ii) The microscopic process attains a stationary distribution which has time reversal symmetry, i.e., $P(x,v) = P(x,-v)$ holds where $P(x,v)$ denotes a stationary probability for the system staying at the coordinate x and the velocity v. That is, any trajectory propagated backward in time also occurs with the equal probability to the original one.

(iii) In a mesoscopic level, we consider Markovian processes of an observable which also has time reversal symmetry.

Under these three assumptions, we can explain why detailed balance holds. Suppose that the probability flows into a state A from another state B. In order for the distribution to be stationary, the decrease of the probability in the state B must be compensated by a flow from a certain state, say C. If the state C is different from A, the decrease of the probability in the state C must be refilled by a flow from still another state. However, existence of such a sequence of flow means that, under a time reversal, the probability flows along the opposite direction, thus breaking the time reversal symmetry of the stationary distribution, that is, the assumption (ii). Therefore, the flow from B to A must be exactly compensated by that from A to B, i.e., detailed balance. Note that the above three assumptions are not mutually exclusive. For example, the assumption (ii) holds only when (i) is satisfied. However, the assumption (i) does not automatically guarantee that (ii) is satisfied. A non-equilibrium steady state with a particle flow is such an example.

In the derivation of detailed balance, the key assumption is that the system attains a stationary distribution having time reversal symmetry. However, as van Kampen pointed out in Ch. VII §2 of the reference[1], there exists a possibility that internal degrees of freedom do not yet reach equilibrium within the timescale of reaction processes. The characteristic timescales of these collective motions could be comparable to the timescales of reaction processes. Then, the reactions under such time-varying circumstances should be described using time-dependent potentials. This possibility gives rise to chemical reactions with some deviation from detailed balance. We will call such reactions chemical ratchets because of the following two reasons: they have similarity to the celebrated Feynman ratchet[2] and involve chemical reactions.

Master equations

A framework of chemical ratchets can be described using the master equation without specifying any details of reactions. Here, we introduce a master equation describing chemical reactions satisfying the basic assumptions for detailed balance. Suppose that the coarse-grained observable takes N states. Let $P_i(t)$ denote the probability of staying at the i-th state at time t, and the equality $\sum_{i=1}^{N} P_i(t) = 1$ holds because of the conservation of probability. We assume that the time evolution of the system is described by the master equation

$$\frac{d}{dt}\boldsymbol{P}(t) = A\boldsymbol{P}(t), \tag{S23}$$

where the i-th component of the vector $\boldsymbol{P}(t)$ is $P_i(t)$, and the component $A_{i,j}$ of the matrix A gives the transition probability from the j-th state to the i-th one. When the distribution $\boldsymbol{P}(t_0)$ at time $t = t_0$ is given, the probability distribution $\boldsymbol{P}(t)$ at time t is uniquely determined for $t > t_0$ solving the master equation Eq.(S23). This means that the time evolution is Markovian.

Because of the conservation of probability, the equality

$$0 = \frac{d}{dt}\sum_{i=1}^{N} P_i(t) = \sum_{i=1}^{N}\sum_{j=1}^{N} A_{i,j}P_j(t), \tag{S24}$$

holds at any time t and for any distribution $\boldsymbol{P}(t)$. Thus, we have the equality

$$0 = \sum_{i=1}^{N} A_{i,j} \tag{S25}$$

for any matrix A describing stochastic processes of coarse-grained observables. This means that the matrix A has an eigenvalue being 0.

Here, we assume that the matrix A satisfies the following conditions.

(i) It does not depend on time.

(ii) It has a non-degenerate eigenvalue being 0. The corresponding eigenvector $\boldsymbol{P}_A^{(0)}$ is the stationary distribution of the system.

(iii) The eigenvector $\boldsymbol{P}_A^{(0)}$ satisfies the property of detailed balance, i.e.,

$$A_{i,j}P_{Aj}^{(0)} = A_{j,i}P_{Ai}^{(0)} \tag{S26}$$

holds for any pair of i and j with $i \neq j$, where $P_{Ai}^{(0)}$ is the i-th component of the stationary state $\boldsymbol{P}_A^{(0)}$.

When the condition (i) holds and the distribution at time t_0 is given, the probability distribution $\boldsymbol{P}(t)$ at time t for $t > t_0$ is uniquely represented by

$$\boldsymbol{P}(t) = \exp\left(A\left(t - t_0\right)\right)\boldsymbol{P}(t_0). \tag{S27}$$

Moreover, when the condition (iii) holds, the matrix A is diagonalizable and its eigenvalues other than 0 are real and negative. Then, any time evolution starting from an initial distribution $\boldsymbol{P}(t_0)$ is given by

$$\boldsymbol{P}(t) = \sum_{l=0}^{N-1} c^{(l)} \exp\left(\lambda_A^{(l)}\left(t - t_0\right)\right)\boldsymbol{P}_A^{(l)}, \tag{S28}$$

where $\boldsymbol{P}(t_0) = \sum_{l=0}^{N-1} c^{(l)}\boldsymbol{P}_A^{(l)}$ is the expansion of the initial distribution using the base which consists of the eigenvectors of the matrix A. Here, we number the eigenvalues of A as $\lambda_A^{(0)} > \lambda_A^{(1)} \geq \cdots \geq \lambda_A^{(N-1)}$, where $\lambda_A^{(0)} = 0$, and denote their corresponding

eigenvectors as $\boldsymbol{P}_A^{(l)}$, respectively. In the following, we define the characteristic timescale of the reaction A by $t_A = 1/\left|\lambda_A^{(1)}\right|$, that is, the slowest relaxation timescale of the reaction A.

The Kolmogorov criterion is a necessary and sufficient condition for a matrix A to have the stationary distribution satisfying detailed balance[3]. It requires that the following equality

$$A_{i_1,i_2}\cdots A_{i_{K-1},i_K}A_{i_K,i_1} = A_{i_K,i_{K-1}}\cdots A_{i_2,i_1}A_{i_1,i_K} \tag{S29}$$

holds for any sequence of transitions between neighboring states in $(i_1, i_2, \cdots, i_{K-1}, i_K, i_1)$ and its inverse transitions, where K is the number of transitions.

We give an example of the matrices A which satisfy the Kolmogorov criterion. Let us consider chemical reactions among states (a set of conformations) of molecules. Here, we think of these states as local minima on the potential (of mean force). They constitute the states in the master equation, and we label them using the indexes $i = 1, 2, \cdots, N$, where the total number of these states is N. Suppose that these local minima are separated by saddles on the potential. We denote the potential of the i-th state as $E_i^{(\mathrm{A})}$ and that of the saddle separating the i-th and j-th states as $E_{i,j}^{(\mathrm{A})}$ where $E_{i,j}^{(\mathrm{A})} = E_{j,i}^{(\mathrm{A})}$ holds for any pair of states (i, j) with $i \neq j$. We define the matrix A describing chemical reactions among these states as follows

$$A_{i,j} = \exp\left(-\left(E_{i,j}^{(\mathrm{A})} - E_j^{(\mathrm{A})}\right)/T\right), \tag{S30}$$

for $i \neq j$ where T is the absolute temperature (we set the Boltzmann constant $k_\mathrm{B} = 1$). We can easily see that the matrix A satisfies the Kolmogorov criterion, thus having the stationary distribution satisfying detailed balance.

Ratchet described by master equations

In the derivation of detailed balance, the key assumption is that the system attains a stationary distribution having time reversal symmetry. When is this assumption guaranteed to hold precisely in nature? For macromolecules such as protein/DNA system *in vitro* or *in vivo* within cells, the system has multiple characteristic timescales covering different orders, e.g., more than $10^{10}-$fold. It is natural that even after some degrees of freedom attain their equilibrium, the other slower degrees of freedom may not, leading to some deviation from detailed balance at least for some timescale of the latter slow degrees of freedom (unless canonical ensemble would be prepared from the very beginning, composed of infinitely large number of copies of proteins and DNA). This mechanism keeps working as long as such degrees of freedom remain non-equilibrium. This is the basic idea of chemical ratchet we propose here along the spirit of van Kampen[1].

In order to investigate this possibility, we present a model of chemical ratchet using the master equation to reveal its mechanism in general without specifying any detailed features of the system. Here, we think of the simplest example of ratchet combining multiple chemical reactions modeled by Eq.(S30). The key to our example is that there exist two kinds of characteristic timescales t_1 and t_2. The timescale t_1 is the characteristic scale for each of the chemical reactions to reach equilibrium. The timescale t_2 is the characteristic scale of stochastic or time-dependent effects originated from external forces or motion of slow degrees of freedom. As the simplest model of ratchets, suppose that a pair of transition matrices A and B are involved in the ratchet, where both of them satisfy the previous conditions (i), (ii) and (iii), and that the reactions described by A and B alternate at every time interval t_{int}. This alternation originates from some stochastic or time-dependent effects, and we assume that $t_A, t_B \sim t_1$ and $t_{\mathrm{int}} \sim t_2$. This alternation keeps driving the system as long as some internal degrees of freedom stay in non-equilibrium.

Here, we think of an ensemble of systems whose reaction processes take place independently with each other. For each system in this ensemble, we can select the origin of time $t = 0$ independently. Therefore, let us define the origin of time for each system to be the time when the reaction by the matrix A starts. Then, the reactions involving A and B are given by $\exp(At_{\mathrm{int}})$ and $\exp(Bt_{\mathrm{int}})$, respectively, for the ensemble of the systems. Thus, one cycle of the total reaction is described by their product as

$$\mathscr{C} = \exp(Bt_{\mathrm{int}})\exp(At_{\mathrm{int}}). \tag{S31}$$

Let us denote the eigenvalues of $\mathscr{C}$ as $\mu_0 > \mu_1 \geq \cdots \geq \mu_{N-1}$, where $\mu_0 = 1$. The net flow $J_{j\to i}$ from the j-th to the i-th states $(j \neq i)$ during one cycle of the ratchet is given by

$$J_{j\to i} = \int_{t_{\mathrm{int}}}^{2t_{\mathrm{int}}} \left(B_{i,j}P_j(t) - B_{j,i}P_i(t)\right)dt + \int_0^{t_{\mathrm{int}}} \left(A_{i,j}P_j(t) - A_{j,i}P_i(t)\right)dt, \tag{S32}$$

where $\boldsymbol{P}(0)$ is an initial distribution.

In Fig. 2 in the paper, we show our model of ratchet where we choose the number of states $N = 3$. In this calculation, we choose as an initial distribution the eigenvector $\boldsymbol{P}_{\mathscr{C}}^{(0)}$ corresponding to the eigenvalue μ_0. Then, the distribution returns to the

initial one after one cycle of the ratchet and the net flow $J_{j\to i}$ does not depend on pairs of neighboring states j and i $(j \neq i)$ as long as the network of the reactions constitutes a circle in general. Therefore, in the following, we denote the net flow as J.

The total reaction given by the matrix $\mathscr{C}$ could be effectively described using the master equation with a constant matrix C as

$$\frac{d}{dt}\boldsymbol{P}(t) = C\boldsymbol{P}(t), \tag{S33}$$

where the matrix C would be given by the equality

$$\exp\left(2Ct_{\text{int}}\right) = \exp\left(Bt_{\text{int}}\right)\exp\left(At_{\text{int}}\right). \tag{S34}$$

Then, the master equation using the matrix C is equivalent to the total reaction composed by the reactions by A and B. The matrix C would be calculated explicitly using the Baker-Campbell-Hausdorff formula (BCH formula)

$$\exp\left(X\right)\exp\left(Y\right) = \exp\left(X + Y + \frac{1}{2}[X,Y] + \frac{1}{12}\left([X,[X,Y]] + [Y,[Y,X]]\right) + \cdots\right), \tag{S35}$$

for matrices X and Y in general, when the BCH formula converges[4]. Here, we use the notation $[X,Y] = XY - YX$. The lowest order approximation $C^{(0)}$ for the matrix C using the BCH formula is given by

$$C^{(0)} = \frac{B+A}{2}. \tag{S36}$$

We can interpret the matrix $C^{(0)}$ as the time average of the ratchet composed by the matrices A and B. Note here that, while the matrices A and B satisfy the Kolmogorov condition, the average $C^{(0)}$ does not necessarily in general. Moreover, the lowest order approximation $C^{(0)}$ does not depend on the timescale of switching t_{int}, while the matrix C in Eq. (S33) generally does.

In the figure, we have displayed the net flow by the matrix $C^{(0)}$. Here, we have chosen as an initial distribution the eigenvector $\boldsymbol{P}^{(0)}_{C^{(0)}}$ corresponding to $\lambda^{(0)}_{C^{(0)}} = 0$. We can see that the net flow by the matrix $C^{(0)}$ provides us with a good approximation for the ratchet as long as $t_{\text{int}} \lesssim \max(t_A, t_B)$ holds, where the net flow increases linearly as a function of the ratio $t_{\text{int}}/\max(t_A, t_B)$.

References

1. van Kampen, N. Stochastic Processes in Physics and Chemistry, Third edition. *Elsevier* (2007).
2. Feynman, R. P. The Feynman Lectures on Physics, Vol. 1, Chapter 46. *Massachusetts, USA: Addison-Wesley* (1963).
3. Kelly, F. P. Reversibility and Stochastic Network, revised edition. *Camb. university press* (2011).
4. Sattinger, D. H. & Weaver, O. L. Lie Groups and Algebras with Applications to Physics, Geometry, and Mechanics. *Springer-Verlag* (2010).

Table 1. Table of symbols

Symbols	definition	dimension<br>M: concentration<br>L: length<br>T: time
$A_{i,j}$	ij component of transition matrix A of master equation corresponding potential A	T^{-1}
$B_{i,j}$	ij component of transition matrix B of master equation corresponding potential B	T^{-1}
D	diffusion constant of one-dimensional diffusion of protein along DNA	L^2T^{-1}
d_0	the average length of DNA covered with protein in one-dimensional diffusion without dissociation from DNA	L
$E_i^{(A)}$	potential value of i state in potential A	energy
$E_{i,j}^{(A)}$	potential value of the saddle between i and j states in potential A	energy
g_0^2	square of tentative constant for convenience defined in Eq. (S8)	ML^{-3}
$J(t)$	net reaction flow at time t	MT^{-1}
J_0	in-coming (out-going if negative) stationary net reaction flow into x_S $(=0)$	MT^{-1}
$J_{j\to i}$	net probability flow from j-state to i-states per unit switching cycle	non-dimensional
K_d	dissociation equilibrium constant	M
k_+^{F-NS}	association rate constant of protein and DNA to form a nonspecific complex	$M^{-1}L^{-1}T^{-1}$
k_-^{F-NS}	dissociation rate constant of a nonspecific complex	T^{-1}
k_+^{F-S}	association rate constant of protein and DNA to form TrpR-*trpO* complex	$M^{-1}T^{-1}$
k_-^{F-S}	dissociation rate constant of TrpR-*trpO* complex	T^{-1}
k_+^{S-NS}	isomerization rate constant of the nonspecific complex to TrpR-*trpO* complex at *trpO*	LT^{-1}
k_-^{S-NS}	isomerization rate constant of TrpR-*trpO* complex to a nonspecific complex at *trpO*	T^{-1}
l	length of DNA segment with nonspecific site	L
M	molar: a unit of concentration defined as mol/litter	M
m_1	fitting parameters in Eq. (11) with m_2, DNA length and d_0	M
m_2	fitting parameters in Eq. (11) with m_1, DNA length and d_0	non-dimensional
n_0	a constant contained in the stationary solution of the differential equation Eq. (8)	ML^{-1}
$n_{NS}(x,t)$	concentration of nonspecific complex per unit length of DNA (1 bp)	ML^{-1}
$n_S(t)$	concentration of TrpR-*trpO* complex, specific complex	M
$n_F^{DNA}(x,t)$	concentration of DNA with empty site at x	M
n_{tot}^{DNA}	total DNA concentration	M
n_{tot}^{prt}	total protein concentration	M
$\boldsymbol{P}(t)$	vector representing the probability distribution of reaction states at time t	non-dimensional
$P_i(t)$	i-th component of the vector $\boldsymbol{P}(t)$, indicating the probability to be i-th state at time t	non-dimensional
q_0	tentative constant for convenience defined in Eq. (S8)	ML^{-2}
t_A, t_B	timescales of reaction expressed by potentials A and B, respectively	T
t_{int}	time between forward and reverse switchings	T
T	temperature with Boltzmann constant set to unity	energy
trpO	operator site for TrpR to repress its downstream gene (*trpR* in this study)	non-dimensional
trpR	gene name for tryptophan repressor	non-dimensional
TrpR	tryptophan repressor protein	non-dimensional
x_S	position of *trpO*	L
α	band intensity at -1T in *trpO* site enhanced by TrpR-*trpO* complex formation in autoradiogram	radioactivity
β	[TrpR] at which half of *trpO* site occupied in the hyperbolic saturation dependence of band intensity	M
Δd	distance between centers of neighboring nonspecific sites on DNA, 1 base pair	L
$\delta(x)$	Dirac's delta function	L^{-1}
